# The association between bullying‐victimisation and sleep disturbances in adolescence: Evidence from a twin study

**DOI:** 10.1111/jsr.13321

**Published:** 2021-03-05

**Authors:** Sania Shakoor, Helena M.S Zavos, Alice M. Gregory, Angelica Ronald

**Affiliations:** ^1^ Wolfson Institute of Preventive Medicine Centre for Psychiatry Queen Mary University of London London UK; ^2^ Department of Psychology Institute of Psychiatry, Psychology and Neuroscience King’s College London London UK; ^3^ Department of Psychology, Goldsmiths, University of London London UK; ^4^ Department of Psychological Sciences Birkbeck, University of London London UK

**Keywords:** behavioural genetics, bullying, insomnia symptoms, sleep quality, twin study

## Abstract

Bullying‐victimisation has been associated with sleep disturbances. This study investigated the degree to which subtypes of bullying‐victimisation in adolescence are linked with sleep disturbances. Genetic and environmental contributions underlying bullying‐victimisation and sleep disturbances were investigated. Participants (3,242–5,076 pairs) from a longitudinal community twin study reported on their bullying‐victimisation at the age of 14 years, and sleep quality and insomnia symptoms at age 16. Regression analyses were used, accounting for the role of individual and family factors. Structural equation twin model fitting was conducted. Bullying‐victimisation was modestly associated with sleep quality and insomnia symptoms (*r *= 0.22–0.23) and a similar strength of associations was found across bullying‐victimisation subtypes (*r *= 0.11–0.22). Bullying‐victimisation, sleep quality and insomnia symptoms were predominantly influenced by genes (25–59%) and non‐shared environments (40–62%). The association between bullying‐victimisation and sleep quality was explained by genetic and non‐shared environmental influences. For insomnia symptoms and sleep quality, the association with bullying‐victimisation was in part explained by a genetic overlap. Associations between bullying‐victimisation and sleep disturbances are not limited to specific aspects of bullying‐victimisation but appear to exist for all subtypes. These findings stimulate research questions regarding the mechanisms underlying these links. For example, could certain heritable traits, such as temperament, increase vulnerability to experiencing sleep disturbances and being bullied? Research on bullying and sleep should aim to take the role of genetic predisposition into account, while also noting that it is not the only causal influence. Understanding more about these pathways could strengthen the development of techniques to prevent these difficulties from occurring.

## 1 INTRODUCTION

Bullying‐victimisation is characterized by repeated hurtful actions between peers where a power imbalance exists (Shakoor et al., 2011). It is associated with psychological and physical difficulties, including emotional and behavioural problems (Arseneault et al., 2010), headaches and sleep disturbances (Gini & Pozzoli, 2013) in childhood and adolescence. Aetiological explorations have found genetic and environmental factors to play pivotal roles (Arseneault, 2018). This has been informative for policymakers and practitioners, as it has provided a greater understanding of the risk bullying‐victimisation poses for well‐being.

An association that still warrants further aetiological exploration is that between bullying‐victimisation and sleep. Important for cognitive and emotional functioning (Walker, 2010), disturbances in sleep have been associated with outcomes frequently seen amongst victims of bullying, such as physical health (Grandner, 2012) and psychological well‐being (Gregory & Sadeh, 2016). It is possible that the fear associated with being bullied and rumination over bullying experiences may interfere with the onset of sleep and contribute towards poor sleep quality and insomnia (Dahl & Lewin, 2002; Harvey, 2002). Evidence supports a robust association between bullying‐victimisation, sleep problems (i.e., difficulties falling or remaining asleep) and insomnia (van Geel et al., 2016), which remains evident after controlling for potential confounders (e.g., emotional and behavioural problems) (Wolke & Lereya, 2014). Bullying‐victimisation is thus an independent risk factor for sleep disturbances and warrants deeper investigation, in particular in adolescence, as this is a period marked by changes in sleep timing and patterns (Crowley, Wolfson, Tarokh, & Carskadon, 2018).

Twin studies have implicated both genetic and environmental factors in bullying‐victimisation (Shakoor et al., 2015) and sleep disturbances (Gregory, 2008). Evidence suggests an overlap in certain correlates, such as socioeconomic deprivation and exposure to violence in the home (Arseneault et al., 2010; Stein et al., 2001). Individual differences in exposure to such things appear to be due to both genetic and environmental influences (Jaffee & Price, 2007). It is thus possible that bullying‐victimisation and sleep disturbances may have shared genetic and environmental influences. Exploring commonalities in environmental and genetic risk factors is important as it can inform strategies applied to dampen the impact of sleep disturbances and promote well‐being. With this in mind, using a genetically sensitive design, the current study aimed to (1) explore the strength of the relationship between bullying‐victimisation and sleep quality and insomnia symptoms during adolescence (after taking family and individual characteristics into consideration) and (2) estimate the extent to which genetic and environmental factors influence these relationships. By using this approach, this study aims to extend the literature on the link between bullying‐victimisation and sleep disturbances and provide an insight into possible aetiological mechanisms.

## 2 METHODS

### 2.1 Sample

Participants belonged to the Twins Early Development Study (TEDS), a longitudinal, general population sample of monozygotic (MZ) and dizygotic (DZ) twins born in England and Wales between 1994 and 1996 (Haworth et al., 2013). TEDS has full ethical approval and written consent was obtained at each point of contact.

Twin reports for 3,527 pairs were obtained at age 14 (*M* = 14.07 years) using postal questionnaires. After exclusions, at age 14, data were available from 3,242 participants, of whom 55% were male, 36% were MZ twin pairs and 31% were opposite sex twin pairs. At age 16, 5,076 twin pairs returned data (*M* = 16.37 years) using postal questionnaires. After exclusions (*n* = 610), the sample was 45% male, 38% were MZ twin pairs and 30% were opposite sex twin pairs. Participants had a mean age of 16.37 years. Exclusion criteria included autism spectrum conditions, genetic syndromes (including fragile X syndrome and cystic fibrosis), chromosomal abnormalities (including Down syndrome and cerebral palsy), extreme perinatal or prenatal complications, and missing first contact or zygosity data. Missing data in twin analyses were dealt with using full information maximum likelihood.

The TEDS sample at ages 14 and 16 years has remained largely representative of the main sample and the UK population (for further details see Haworth et al., 2013; Ronald et al., 2014). Zygosity was ascertained through a combination of DNA testing and parent report of twin resemblance, which has been found to be over 90% accurate when compared to DNA testing alone (Price et al., 2000).

### 2.2 Measures

#### 2.2.1 Age 14: bullying‐victimisation

Bullying victimisation was assessed using the Multidimensional Peer Victimisation Scale (Mynard & Joseph, 2000). Consisting of 16 self‐report items, the scale captured four subscales of bullying‐victimisation: physical abuse, verbal abuse, social manipulation and property damage. Each subscale consisted of four items and was created in line with previous research (Mynard & Joseph, 2000). Summing across all items captured a total composite measure of bullying‐victimisation. Higher scores indicated higher levels of bullying‐victimisation. High internal reliability of scales and the total composite measure was evident in our sample (Table 1).

**TABLE 1:** Means and standard deviations by sex and zygosity for bullying‐victimisation sleep quality and insomnia symptoms

	Total	Male	Female	MZ	DZ	Score			Cronbach	ANOVA					
	*M* (*SD*)	*M* (*SD*)	*M* (*SD*)	*M* (*SD*)	*M* (*SD*)	Range	Skew	Kurtosis	Α	Sex	Zyg	Sex*Zyg		R^2^	*N*
Bullying‐victimisation															
Total	7.61 (7.18)	8.47 (7.73)	6.92 (6.61)	7.48 (7.15)	7.69 (7.19)	0–32	−0.01	2.20	0.90	0.00	0.48	0.51		0.01	3,208
Physical	1.02 (1.85)	1.68 (2.23)	0.49 (1.25)	1.00 (1.89)	1.04 (1.83)	0–8	1.17	2.91	0.80	0.00	0.75	0.21		0.11	3,208
Verbal	3.31 (2.84)	3.62 (2.82)	3.07 (2.83)	3.20 (2.82)	3.38 (2.85)	0–8	0.35	1.72	0.84	0.00	0.13	0.74		0.01	3,208
Social manipulation	1.90 (2.29)	1.53 (2.08)	2.20 (2.40)	1.91 (2.27)	1.90 (2.30)	0–8	0.30	1.70	0.80	0.00	0.78	0.10		0.02	3,208
Property damage	1.37 (1.98)	1.65 (2.19)	1.15 (1.76)	1.37 (1.98)	1.38 (1.98)	0–8	0.65	2.13	0.79	0.00	0.80	0.66		0.01	3,208
														
Sleep quality	5.52 (2.74)	5.18 (2.57)	5.81 (2.85)	5.40 (2.70)	5.58 (2.76)	0–21	0.13	3.07	0.76	0.00	0.02	0.70		0.01	3,732
Insomnia symptoms	3.81 (4.26)	3.27 (3.88)	4.27 (4.52)	3.61 (4.17)	3.92 (4.31)	0–28	0.35	2.67	0.89	0.00	0.01		0.85	0.02	3,721

Note

Means and standard deviation reported prior to transformation. MZ: monozygotic; DZ: dizygotic twins. Analyses of variances were performed using one random member of each twin pair. Sex: *p*‐value associated with the effect of sex on the means; Zyg: *p*‐value associated with the effect of zygosity on the means; Sex*Zyg: *p*‐value associated with the effects of the interaction between sex and zygosity on the means; R^2^: proportion of the total variance explained by sex and zygosity; *N*: number of one randomly selected individuals from each twin pair. For sleep quality, higher scores indicate greater problems.

#### 2.2.2 Age 16: sleep

Sleep quality was assessed using the Pittsburgh Sleep Quality Index (PSQI; Buysse et al., 1989). The measure consisted of 17 self‐report items, which assessed sleep quality in the past month by enquiring about duration of sleep, amount of time spent in bed, daytime disruption, sleep medication use, and whether sleep had been disrupted by factors such as struggling to fall and stay asleep. Higher scores indicated poorer sleep quality. The PSQI displayed good internal consistency (Table 1) in the current sample.

Insomnia symptoms were assessed using the Insomnia Severity Index (ISI; Bastien et al., 2001). Consisting of seven self‐report items, the measure assessed symptoms of insomnia, such as sleep problems interfering with daily functioning. Higher scores represented greater insomnia symptoms. The ISI had good internal consistency (Table 1) in the current sample.

#### 2.2.3 Age 12: covariates

Covariates were selected based on previous research (Kochel et al., 2012; Stein et al., 2001). These included bullying‐victimisation, cognitive ability, behavioural problems, emotional problems (including symptoms of anxiety), depression, home chaos, parental discipline and socioeconomic status. It should be noted that behavioural problems include some items that encompass emotional problems; therefore, these two scales were not entirely independent of each other. Details and descriptive statistics of these measures are reported in Table S1.

### 2.3 Statistical analyses

Analyses were performed using statistical packages STATA 12 (StataCorp. Stata Statistical Software: Release 14. College Station) and Open Mx (Boker et al., 2011). Open Mx uses the method of maximum likelihood estimation and is widely used for analysing genetically sensitive data. In line with standard behavioural genetics procedures, the effects of sex and age were regressed on the phenotypes. Further analyses were conducted using the remaining residuals for each phenotype. Any skewed data were transformed using square root transformation techniques. Analyses were performed as follows: (1) using linear and multivariate regression models we examined the association between bullying‐victimisation and sleep whilst taking into account a series of covariates (listed above); (2) we tested the degree of twin similarity on the measures for monozygotic (MZ) and dizygotic (DZ) males and females and DZ opposite sex (DZOS); (3) we used univariate structural equation models to estimate the contributions of genetic and environmental influences to bullying‐victimisation and sleep measures; and (4) we ran bivariate correlated factors models to examine the overlap between genetic and environmental influences on bullying‐victimisation and sleep.

### 2.4 The twin design

The twin design uses MZ and DZ twin pairs to estimate variation in a phenotype, or covariation between phenotypes, that is attributable to genetic and environmental influences. Within‐pair similarities for MZ and DZ twin pairs were examined to establish the role of genetic and environmental influences based on the assumption that: (1) MZ twin pairs share 100% of their segregating genes and DZ twin pairs share on average 50%; (2) MZ and DZ twin pairs share environmental factors that make both twins in the same family more alike (“shared environment”); and (3) exposure to environmental factors that are experienced differently or are specific to the individual (“non‐shared environment”) contributes towards differences between MZ and DZ twin pairs. This includes measurement error (Rijsdijk & Sham, 2002). Structural equation modelling was employed to establish the relative importance of additive genetic (A), shared environment (C) and non‐shared environmental influences (E) (Rijsdijk & Sham, 2002) (Appendix S1 for more details).

## 3 RESULTS

### 3.1 Descriptive statistics

Analyses revealed significant mean sex effects for all scales (Table 1). Relative to females, males reported higher levels of bullying‐victimisation (with the exception of social manipulation). Females reported poorer sleep quality and greater insomnia symptoms than did males. A main effect for zygosity was observed, whereby DZs reported higher levels of sleep problems in comparison to MZs. However, the combined effect of sex and zygosity on the means for all phenotypes was small (*R* ^2^ = 0.00–0.11).

### 3.2 Phenotypic associations between bullying‐victimisation subscales at age 14 and sleep at age 16

Phenotypic correlations between bullying‐victimisation subtypes and sleep were small but significant (range *r* = 0.12–0.24). There were no differences between the magnitude of the associations between the sleep phenotypes and the different bullying‐victimisation subtypes (Table 2). Associations between bullying‐victimisation, sleep quality and insomnia symptoms remained significant after accounting for possible individual and family confounders, including emotional and behavioural problems, socioeconomic status (SES) and parental discipline (Table 3). The associations were higher amongst females; however, the differences were non‐significant as compared to males. The associations between bullying‐victimisation subtypes, sleep quality and insomnia symptoms became non‐significant once confounders were taken into account.

**TABLE 2:** Phenotypic correlations between bullying‐victimisation subscales, sleep quality and insomnia symptoms

	Total		Male		Female	
	Sleep quality r (95% CI)	Insomnia symptoms r (95% CI)	Sleep quality r (95% CI)	Insomnia symptoms r (95% CI)	Sleep quality r (95%CI)	Insomnia symptoms r (95% CI)
*Bullying‐victimisation*						
Total bullying‐victimisation	0.22 (0.18/0.25)	0.22 (0.18/0.25)	0.19 (0.14−0.24)	0.19 (0.14−0.24)	0.24 (0.20−0.29)	0.23 (0.19−0.27)
Physical	0.13 (0.09/0.16)	0.14 (0.11/0.18)	0.12 (0.07−0.17)	0.12 (0.07−0.18)	0.15 (0.10−0.19)	0.17 (0.12−0.22)
Verbal	0.21 (0.17/0.24)	0.19 (0.16/0.23)	0.16 (0.11−0.21)	0.17 (0.11−0.22)	0.24 (0.20−0.29)	0.21 (0.17−0.26)
Social manipulation	0.17 (0.14/0.21)	0.16 (0.13/0.20)	0.17 (0.11−0.21)	0.17 (1.2−0.23)	0.18 (0.13−0.22)	0.16 (0.11−0.20)
Property damage	0.16 (0.13/0.20)	0.16 (0.13/0.19)	0.16 (0.11−0.21)	0.15 (0.10−0.21)	0.17 (0.12−0.22)	0.17 (0.12−0.21)

Note

Correlations taken from the constrained saturated bivariate model where phenotypic correlations and twin correlations within and across measures were estimated. R: Pearson's correlation; CI: confidence interval. Bullying‐victimisation measured at age 14 years; sleep quality and insomnia symptoms measured at age 16.

**TABLE 3:** Associations between bullying‐victimisation, sleep quality and insomnia symptoms

	Total		Males		Female	
	Unadjusted	Adjusted	Unadjusted	Adjusted	Unadjusted	Adjusted
	β (95% CI)	β (95% CI)	β (95% CI)	β (95% CI)	β (95% CI)	β (95% CI)
*Dependent variable: Sleep quality*						
Total bullying‐victimisation	0.21 (0.16, 0.26)	0.19 (0.13, 0.25)	0.19 (0.12, 0.26)	0.18 (0.09, 0.27)	0.26 (0.20, 0.33)	0.20 (0.12, 0.28)
*Bullying‐victimisation subtypes*						
Physical	0.06 (0.01, 0.11)	−0.07 (−0.13, 0.01)	0.10 (0.04, 0.16)	−0.04 (−0.13, 0.06)	0.14 (0.04, 0.23)	−0.07 (−0.19, 0.05)
Verbal	0.20 (0.15, 0.25)	0.15 (0.08, 0.22)	0.18 (0.11, 0.25)	0.09 (−0.02, 0.20)	0.25 (0.19, 0.32)	0.18 (0.08, 0.28)
Social manipulation	021 (0.16, 0.26)	0.08 (0.02, 0.15)	0.19 (0.11, 0.26)	0.12 (0.02, 0.23)	0.20 (0.14, 0.27)	0.05 (−0.04, 0.14)
Property damage	0.14 (0.09, 0.19)	0.06 (−0.01, 0.13)	0.15 (0.08, 0.21)	0.06 (−0.04, 0.13)	0.17 (0.10, 0.24)	0.05 (−0.02, 0.14
*Dependent variable: Insomnia symptoms*						
Total bullying‐victimisation	0.21 (0.16, 0.25)	0.16 (0.10, 0.22)	0.17 (0.10, 0.23)	0.12 (0.03, 0.20)	0.27 (0.21, 0.34)	0.20 (0.12, 0.29)
*Bullying‐victimisation subtypes*						
Physical	0.06 (0.01, 0.11)	−0.04 (−0.12, 0.03)	0.09 (0.03, 0.15)	−0.02 (−0.12, 0.07)	0.18 (0.08, 0.27)	−0.04 (−0.17, 0.09)
Verbal	0.18 (0.13, 0.22)	0.10 (0.02, 0.17)	0.16 (0.09, 0.23)	0.07 (−0.04, 0.18)	0.23 (0.16, 0.29)	0.11 (0.01, 0.21)
Social manipulation	0.22 (0.17, 0.26)	0.09 (0.02, 0.16)	0.18 (0.11, 0.25)	0.09 (−0.02, 0.20)	0.21 (0.14, 0.28)	0.09 (−0.01, 0.18)
Property damage	0.14 (0.09, 0.19)	0.05 (−0.02, 0.11)	0.13 (0.07, 0.19)	0.04 (−0.05, 0.14)	0.18 (0.11, 0.26)	0.05 (−0.05, 0.15)

Note

β: standardized beta coefficient; CI: confidence interval. Each model adjusts for all individual and family factors simultaneously in one model. Individual and family factors measured at age 12 years include bullying‐victimisation, behavioural problems, emotional problems, depression, general cognition, home chaos, parental discipline and socioeconomic status. Analyses within each subtype of bullying‐victimisation adjust for the remaining subtypes of bullying‐victimisation in addition to the individual and family factors. Bullying‐victimisation measured at age 14 years; sleep quality and insomnia symptoms measured at age 16.

### 3.3 Univariate analysis

MZ correlations (Table S2) were higher than DZ correlations, suggesting genetic influences on bullying‐victimisation and sleep disturbances. Sex differences in variance components were indicated by a different pattern of correlations between MZ and DZ pairs in males and females. Structural equation model fitting analysis confirmed these initial observations (Table S3). Equating A, C and E estimates across sexes led to significant deterioration in fit for all variables, with the exception of sleep quality. Parameter estimates for the best fitting univariate models are shown in Table 4. Significant genetic influences were shown for all variables in males (all bullying measures, 25%–59%; insomnia symptoms, 34%) and females (all bullying measures, 11%–39%; insomnia symptoms, 42%). Shared environmental influences were evident for some bullying measures, including total bullying‐victimisation, and were greater in females (3%–31%) than males (2%–19%), except for physical bullying. Shared environmental influences were not significantly evident for insomnia symptoms (3%–4%). Non‐shared environmental influences were significant for all bullying measures (40%–61%) and insomnia symptoms (56%–62%) in males and females. No significant sex differences were observed for sleep quality; 42% of variances in sleep quality was attributable to genetic factors, 58% to non‐shared environmental factors, and no significant contribution of shared environmental factors was observed.

**TABLE 4:** Parameter estimates for best fitting univariate models

	Parameter estimates of best fitting model								
	Total sample			Males			Females		
Measure	A (95% CI)	C (95% CI)	E (95% CI)	A (95% CI)	C (95% CI)	E (95% CI)	A (95% CI)	C (95% CI)	E (95% CI)
Total bullying	–	–	–	0.59 (0.54, 0.65)	0.00 (0.00, 0.03)	0.40 (0.35, 0.45)	0.37 (0.22, 0.52)	0.23 (0.08, 0.36)	0.40 (0.36, 0.45)
Physical	–	–	–	0.32 (0.15, 0.46)	0.18 (0.05, 0.32)	0.51 (0.45, 0.57)	0.39 (0.29, 0.46)	0.03 (0.01, 0.10)	0.59 (0.53, 0.65)
Verbal	–	–	–	0.59 (0.50−0.64)	0.00 (0.00−0.09)	0.41 (0.36−0.46)	0.36 (0.20−0.54)	0.18 (0.02−0.32)	0.45 (0.41−0.51)
Soc manipulation			–	. 25 (0.11, 0.53)	0.19 (0.01, 0.30)	0.56 (0.50, 0.63)	0.16 (0.02, 0.30)	0.31 (0.07, 0.43)	0.53 (0.47, 0.58)
Property damage				0.41 (0.24, 0.49)	0.02 (0.01, 0.16)	0.57 (0.01, 0.63)	0.11 (0.01, 0.30)	0.28 (0.12, 0.40)	0.61 (0.55, 0.66)
Sleep quality	0.42 (0.37, 0.46)	0.00 (0.00, 0.04)	0.58 (0.54, 0.61)	–	‐	‐	‐	‐	‐
Insomnia symptoms	‐	‐	‐	0.34 (0.17−0.43)	0.04 (0.00−0.18)	0.62 (0.56−0.68)	0.42 (0.28−0.49)	0.03 (0.00−0.14)	0.56 (0.50−0.61)

Note

Total bullying: total bullying‐victimisation; Soc manipulation: social manipulation; A: additive genetic influences; C: shared environmental influences; E: non‐shared environmental influences; CI: confidence intervals

### 3.4 Bivariate twin model‐fitting results

Correlations between bullying‐victimisation (composite score) and sleep quality and insomnia symptoms were significant (*r*ph = 0.19–0.24, Table 2). Due to the associations between bullying‐victimisation subtypes and sleep disturbances not remaining after confounders were taken into consideration, we focus on the relationship between the total bullying‐victimisation and sleep disturbances in the bivariate twin model‐fitting analyses.

Cross‐twin cross‐trait (CTCT) correlations are shown in Table 5. Due to the evidence of the effect of sex differences on variables of interest we fitted a bivariate heterogeneity (sex differences) model (fit statistics presented in Table S4). CTCT correlations suggested that in addition to genetic influences, shared environmental influences may account for some of the covariance between traits (MZM and DZM CTCT correlations were similar). Equating parameters across sexes (bivariate homogeneity model) led to a significant deterioration in fit (Table S4). However, as patterns of genetic and environmental factors were similar across sexes (see Figures S1a and S1b), here we focus on the results from the homogeneity model.

**TABLE 5:** Cross‐twin cross‐trait correlations and proportion of phenotypic correlation due to genetic and environmental factors for relationship between total bullying‐victimisation, sleep quality and insomnia

	Total bullying: Sleep quality (95% CI)	Total bullying: Insomnia symptoms (95% CI)
*Cross‐twin cross‐trait correlations*		
MZ	0.17 (0.12−0.21)	0.18 (0.14−0.22)
DZ	0.11 (0.07−0.16)	0.13 (0.09−0.17)
MZM	0.10 (0.03−0.17)	0.14 (0.07−0.21)
DZM	0.11 (0.02−0.19)	0.13 (0.05−0.22)
MZF	0.22 (0.16−0.27)	0.21 (0.15−0.27)
DZF	0.15 (0.08−0.22)	0.16 (0.09−0.23)
DZOS	0.10 (0.04−0.16)	0.11 (0.05−0.17)
*Proportion of phenotypic correlation due to genetic and environmental factors: total sample*		
A	0.50 (0.27/0.87)	0.62 (0.39/0.98)
C	0.26 (−0.05/0.41)	0.24 (−0.06/0.39)
E	0.24 (0.09/0.40)	0.14 (−0.02/0.30)
*Proportion of phenotypic correlation due to genetic and environmental factors: males*		
A	0.52 (0.17/0.90)	0.67 (0.63/0.92)
C	0.05 (−0.06/0.19)	0.13 (−0.04/0.61)
E	0.43 (0.14/0.74)	0.21 (0.08/0.51)
*Proportion of phenotypic correlation due to genetic and environmental factors: females*		
A	0.39 (−0.12/1.00)	0.47 (−0.03/0.58)
C	0.48 (−0.05/0.90)	0.45 (−0.18/0.87)
E	0.13 (0.02/0.25)	0.09 (−0.11/0.28)

Note

Correlations taken from the constrained saturated bivariate model where phenotypic correlations and twin correlations within and across measures were estimated. MZ: monozygotic; DZ: dizygotic; MZM: monozygotic male; DZM: dizygotic male; MZF: monozygotic female; DZF: dizygotic female; DZOS: dizygotic opposite sex; A: additive genetic; C: shared environment; E: non‐shared environment

**FIGURE 1:**
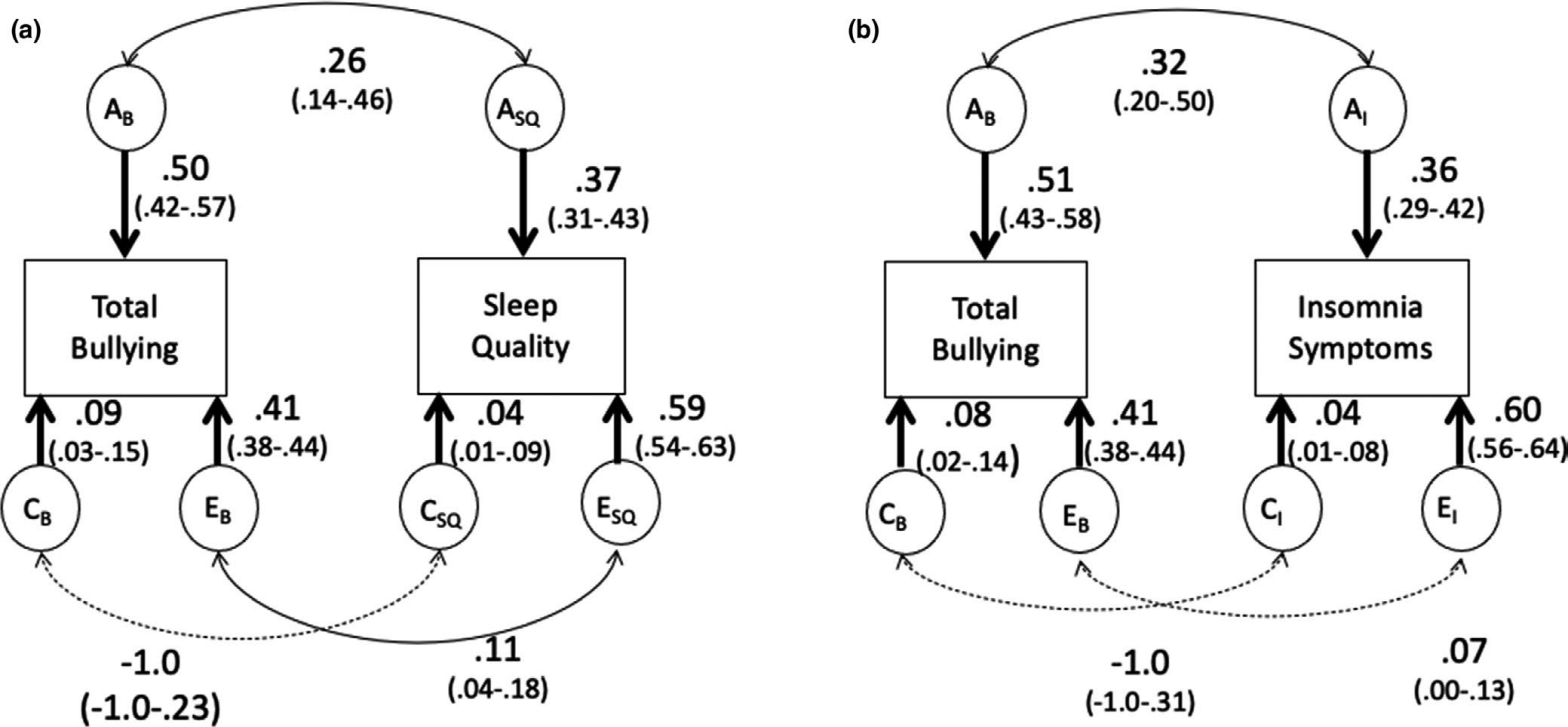
(a) Correlated factors solution of relationship between bullying‐victimisation and sleep quality. A_B_: additive genetic influences on bullying‐victimisation; A_SQ_: additive genetic influences on sleep quality; C_B_: shared environmental influences on bullying‐victimisation; C_SQ_: shared environmental influences on sleep quality; E_B_: non‐shared environmental influences on bullying‐victimisation; E_SQ_: non‐shared environmental influences on sleep quality. Non‐significant parameters indicated with doted lines. (b) Correlated factors solution of relationship between bullying‐victimisation and insomnia symptoms. A_B_: additive genetic influences on bullying‐victimisation; A_I_: additive genetic influences on insomnia symptoms; C_B_: shared environmental influences on bullying‐victimisation; C_I_: shared environmental influences on insomnia symptoms; E_B_: non‐shared environmental influences on bullying‐victimisation; E_I_: non‐shared environmental influences on insomnia symptoms. Non‐significant parameters indicated with doted lines

Figure 1a presents the results of the bivariate analysis of the association between total bullying‐victimisation and sleep quality. The relationship between total bullying‐victimisation and sleep quality was in part due to overlapping genes (rA = 0.26; 95% confidence interval [CI], 0.14–0.46). A significant correlation between non‐shared environmental influences was also observed (rE = 0.11 95%; CI, 0.04–0.18). Genetic and non‐shared environmental influences both significantly explained the phenotypic correlation (50% and 24%, respectively; Table 5). Shared environmental influences were non‐significant.

Figure 1b presents the results of the bivariate model of the association between total bullying‐victimisation and insomnia symptoms. Covariation between total bullying‐victimisation and insomnia symptoms was due to genetic influences (rA = 0.32; 95% CI, 0.20–0.50). Although a non‐shared environment accounted for 41%–60% of the variance in bullying‐victimisation and insomnia symptoms, these influences were not significantly correlated (rE = 0.07; 95% CI, 0.00–0.13). As such, there was not significant overlap in non‐shared environmental influences between bullying victimisation and insomnia. Rather non‐shared environments that are specific to bullying and specific to insomnia were contributing towards each phenotype. Genetic influences did account for a significant proportion of the phenotypic correlation (Table 5; 62%).

## 4 DISCUSSION

### 4.1 Childhood bullying‐victimisation and sleep disturbances

This study found bullying‐victimisation at age 14 years to be similarly associated with sleep quality and insomnia symptoms at age 16. Associations remained significant for the composite score of bullying‐victimisation after controlling for individual and family characteristics. Our results are consistent with previous reports from population‐based studies in children and adolescents (van Geel et al., 2016). Our findings extend past work by showing that the association between bullying‐victimisation and troubled sleep is not specific to a particular type of bullying‐victimisation. The overall experience of bullying‐victimisation is associated with poorer sleep quality and insomnia symptoms.

Our findings are consistent with the idea that exposure to threat, assessed here as bullying‐victimisation, can contribute to sleep disturbances (Dahl & Lewin, 2002). It is possible that cognitive processes could mediate this association. For example, victims of bullying have been found to ruminate more about their experiences in comparison to their non‐bullied peers (Hampel et al., 2009). The process of rumination is observed amongst people with insomnia symptoms who report that worrying, intrusive thoughts or a racing mind contribute towards their difficulties with sleep (Harvey, 2002). The heightened preoccupation with past events amongst victims of bullying could be an underlying mechanism explaining the covariation between bullying‐victimisation and insomnia symptoms.

### 4.2 The heritability of bullying‐victimisation and sleep disturbances

In concurrence with previous twin studies, both sleep and bullying‐victimisation were heritable, suggesting that adolescents have genetic predispositions that increase their vulnerability to being bullied and experiencing sleep problems (Gregory, 2008; Shakoor et al., 2015). Our study also found significant non‐shared influences for all phenotypes. This indicates that after taking genetic vulnerabilities into account, it is environments that are specific to the individual, such as individual‐specific stressful life events, that contribute towards their risk of being bullied and having sleep disturbances. Shared environmental influences were evident for some bullying measures and tended to be greater in females than males. This suggests that the environmental experiences that make family members more alike, which could potentially include socioeconomic deprivation and exposure to violence in the home, may be influential in shaping vulnerability to most types of bullying‐victimisation. To our knowledge, this is the first study to estimate genetic and environmental influences on the subtypes of bullying‐victimisation, and although all subtypes share a commonality in their genetic and non‐shared environmental influences, the magnitude of effect of the shared environmental influences was significantly higher in certain types of bullying‐victimisation.

### 4.3 Aetiology underlying the association between bullying‐victimisation and sleep disturbances

The association between bullying‐victimisation at age 14 years and insomnia symptoms and sleep quality at age 16 was in part explained by overlapping genetic influences. Furthermore, consistent with the possibility of gene–environment correlation, our finding of genetic overlap indicates individuals may have inherent genetic predispositions that influence vulnerability to being bullied and experiencing sleep disturbances. For example, early emotional problems increase the risk of bullying‐victimisation (Arseneault et al., 2010). Anxious and depressed individuals may appear less capable of negotiating conflicts or of defending themselves, and are thus viewed as easy targets for abuse from peers. As emotional problems are in part heritable (Bartels et al., 2011) and associated with sleep disturbances (Gregory, 2008), it is theoretically possible that genetic influences underlying the relationship between bullying‐victimisation and sleep disturbances are explained by co‐occurring emotional problems. We explored this in our data and found that the phenotypic association between bullying‐victimisation and sleep disturbances remained after controlling for emotional problems, suggesting that other mechanisms may be at play (Table 3).

Our finding of genetic overlap between bullying‐victimisation and sleep disturbances should be explored further. For example, genetic variants identified from recent genome‐wide association studies as being associated with insomnia (Jansen et al., 2019) and sleep length in children and adults (Jones et al., 2016; Marinelli et al., 2016) could be explored for their potential associations with vulnerability to bullying‐victimisation. Furthermore, using polygenic risk score analyses, information from genome‐wide data could be collated to see whether there is an overlap between the common risk alleles associated with bullying‐victimisation and sleep disturbances.

### 4.4 Limitations and strengths

Although we measured bullying‐victimisation before sleep quality and insomnia symptoms, we were unable to control for earlier insomnia symptoms and sleep quality. This study therefore does not show that bullying‐victimisation is associated with an increase in disturbed sleep over time. The association between sleep disturbances and bullying‐victimisation may be bidirectional, with sleep disturbances increasing the likelihood of bullying‐victimisation and vice‐versa, or influenced by a third underlying factor. Data on sleep disturbances in adolescents were only available at age 16 years; as such, we were unable to test for a bidirectional relationship.

We used self‐reports of bullying‐victimisation. It is possible that adolescents at age 14 may not be comfortable reporting bullying‐victimisation; however, self‐reports of bullying‐victimisation at a younger age of 12 have been found to be reliable and comparable to parent reports (Shakoor et al., 2011). We also relied on questionnaire measures of sleep disturbances. Polysomnography and actigraphy measures would provide additional objective perspectives on aspects of sleep (Gregory & Sadeh, 2016), although this form of measurement is often not feasible in large samples. Furthermore, the PSQI and ISI are well‐established and validated instruments (Bastien et al., 2001; Buysse et al. 1989). Our measures of sleep focused on sleep quality and insomnia symptoms. Other sleep disturbances, such as parasomnias, have been found to be associated with bullying‐victimisation (Wolke & Lereya, 2014) and investigation into the aetiology of further associations would be informative. Furthermore, we observed substantial overlap between our measures of sleep quality and insomnia symptoms (r = 0.69; *p* <.01). This was expected, although needs to be considered when interpreting the results of the study. We decided to include both measures in this report because they have distinct properties. Indeed, it is possible to report poor sleep quality without reporting insomnia. By including both measures we are capturing a wider range of sleep behaviours.

All of our measures were self‐report, which means it is possible that there was potential bias due to shared methods variance. We explored this in our data by using the Harman's single factor test. Using factor analyses we checked if one single factor emerged or whether a general factor explained the majority of the covariance amongst the measures. It is argued that this general factor captures common methods variance (Podsakoff et al., 2003). Analyses revealed nine factors explaining 59.22% of the variance. The primary unrotated factor captured only 21.42% of the variance (Table S5). These results do not support the presence of common methods variance. We therefore consider the potential effects of common method variance to be non‐substantial.

Despite these limitations, this study's strengths lie in its genetically informative twin design study, which allowed decomposition of the relationship between bullying‐victimisation and sleep disturbances into genetic and environmental influences. Moreover, the large sample size and multiple dimensions of bullying‐victimisation allowed us to conduct novel analyses on the relationship between sleep disturbances and specific types of bullying‐victimisation.

## 5 CONCLUSIONS

Bullying‐victimisation at the age of 14 years is associated with sleep disturbances at age 16. These associations were driven by genetic and non‐shared environmental risk factors that are partly common to both. Thus, rather than viewing bullying‐victimisation as a purely environmental experience that can trigger later sleep disturbances, practitioners and researchers may benefit from focusing on inherited characteristics (e.g., mental health problems and temperament) that may make individuals jointly vulnerable to being bullied and experiencing sleep disturbances.

## 6 ACKNOWLEDGEMENTS

The authors thank the participants of the Twins Early Development Study for making this research possible. Thank you also to Professor Robert Plomin, Andrew McMillan, Francesca Lewis, Louise Webster, Neil Harvey and Rachel Ogden.

## CONFLICT OF INTEREST

Alice Gregory is an advisor for a project sponsored by Johnson's Baby. She has written the books Nodding Off (Bloomsbury Sigma, 2018) and The Sleepy Pebble (Flying Eye Books, 2019). She is a regular contributor to BBC Focus magazine and has contributed to other outlets (such as The Conversation, The Guardian and Balance Magazine). She occasionally receives sample products related to sleep (e.g., blue light blocking glasses) and has given a paid talk to a business. Angelica Ronald has written for the National Childbirth Trust. Sania Shakoor is a trustee for the anti‐bullying charity Kidscape.

## AUTHOR CONTRIBUTIONS

All authors have made substantial contributions to the conception, design, analysis and interpretation of data for the work presented. They contributed to the drafting of the manuscript and revising it critically for intellectual content.

7

## Supporting information

Supplementary Material

## Data Availability

Research data are not shared.
