# Supplementary material for: The association between bullying‐victimisation and sleep disturbances in adolescence: Evidence from a twin study
Source: J Sleep Res. 2021 Mar 5;30(5):e13321. doi: 10.1111/jsr.13321 (PMC11475508; doi:10.1111/jsr.13321)
Supplement: Supplementary file 1 — Supplementary Material [file JSR-30-e13321-s001.docx]

***Supplementary methods S1: The twin design***

Structural equation modelling was employed to establish the relative importance of additive genetic (A), shared environment (C) and non-shared environmental influences (E) contributing to a phenotype (Rijsdijk & Sham, 2002). If the pattern of MZ and DZ correlations suggested possible sex differences, heterogeneity and homogeneity ACE models were run and the relative fit of these models was assessed. Heterogenetiy models estimate A, C and E separately for males and females. In the homogeneity model A, C and E are equated across males and females. The homogeneity model is compared to the less constrained heterogeneity model. If the homogeneity model did not lead to a decline in fit we chose this model in line with rules of parsimony. Fit statistics provided by Open Mx for raw data modelling is minus twice the log likelihood (−2LL) of the observations.

This technique further extends to bivariate analyses, by exploring the covariation between phenotypes. The extent to which genetic and environmental influences are correlated between phenotypes is calculated by estimating the genetic correlations (r^a^), shared environment correlations (r^c^) and non-shared environment correlations (r^e^). In addition to the genetic and environmental correlations, it is also possible to calculate the proportion of the phenotypic correlation due to genes or environment. As with the univariate analysis, two genetic models were fitted to the data. First a quantitative genetic model in which parameters of A, C and E and correlations between bullying and sleep were estimated separately for males and females. The specification of the model was in line with methods used to allow for the inclusion of opposite sex pairs (Neale, Roysamb, & Jacobson, 2006). A homogeneity model was then fit to the data in which one set of A, C and E parameters across the sexes are estimated. The differences in fit of these models were evaluated by likelihood ratio testing.

Table S1: Measures of confounders in the sample

|  | **Measure** | **Informant** | **Mean (SD)** | **Range** | **Reference Citations** |
| --- | --- | --- | --- | --- | --- |
| **Age-12 Confounders** |  |  |  |  |  |
| **Individual Factors** |  |  |  |  |  |
| **Bullying victimisation** | Multidimensional Peer Victimisation Scale | Self | 7.80 (7.35) | 0-32 | (Mynard & Joseph, 2000) |
| **Behaviour problems** | SDQ (Strengths and Difficulties Questionnaire) | Parent | 7.01 (5.04) | 0-32 | (Goodman, 1997) |
| **Emotional Problems** | SDQ (Strengths and Difficulties Questionnaire) | Parent | 1.81 (1.91) | 0-10 | (Goodman, 1997) |
| **Depression** | MFQ (Moods and Feelings Questionnaire) | Parent | 1.80 (2.81) | 0-25 | (Angold et al., 1995) |
| **General IQ** | WISC-III-PI Information Multiple-Choice. | Self | 0.01 (1.00) | -4.05-2.78 | (Kaplan, Fein, Kramer, Delis, & Morris, 1999) |
| **Family factors** |  |  |  |  |  |
| **Home chaos** | Confusion, Hubbub and Order Scale (CHAOS) | Parent | 3.31 (1.83) | 0-10 | (Matheny, Wachs, Ludwig, & Phillips, 1995) |
| **Parental discipline** | Parental discipline | Parent | 2.14 (1.14) | 0-7 | (Deater-Deckard, Dodge, Bates, & Pettit, 1998) |
| **Socioeconomic status** | Socioeconomic status: parental education, occupation, and family income. | Parent | 0.10 (0.99) | -2.60-2.65 |  |

Table S2: Monozygotic and dizygotic twin correlations

|  | Total bullying | Physical | Verbal | Social manipulation | Property damage | Sleep quality | Insomnia |  |
| --- | --- | --- | --- | --- | --- | --- | --- | --- |
| MZM | .60 (.54-.64) | .50 (.44-.56) | .59 (.53-.64) | .45 (.38-.52) | .44 (.37-.50) | .39 (.32-.45) | .39 (.32-.46) | |
| DZM | .33 (.24-.40) | .31 (.23-.39) | .32 (.24-.40) | .27 (.19-.35) | .21 (.12-.30) | .15 (.06-.23) | .19 (.11-.27) |  |
| MZF | .60 (.55-.64) | .44 (.37-.49) | .54 (.49-.59) | .48 (.43-.53) | .39 (.33-.45) | .48 (.43-.53) | .45 (.40-.50) |  |
| DZF | .41 (.34-.47) | .15 (.06-.23) | .36 (.29-.43) | .37 (.30-.44) | .34 (.26-.41) | .19 (.11-.26) | .21 (.14-.28) |  |
| DZOS | .24 (.18-.30) | .09 (.02-.15) | .22 (.16-.28) | .16 (.09-.22) | .18 (.12-.24) | .19 (.14-.24) | .15 (.09-.20) |  |

Note: Twin correlations taken from the constrained saturated model. MZM=Monozygotic male; DZM=dizygotic male; MZF= Monozygotic female; DZF=dizygotic female; DZOS=dizygotic opposite sex

Table S3: Fit statistics for univariate ACE models

|  |  | Model Fit | | | | | |
| --- | --- | --- | --- | --- | --- | --- | --- |
| Measure | Model | -2LL | df | LRT | Δdf | AIC | P |
| Total bullying | Saturated | 22145.88 | 6388 | - | - | 9369.89 | - |
|  | Constrained Saturated | 22153.70 | 6402 | 7.81 | 14 | 9349.70 | 0.90 |
|  | **Heterogeneity *** | **22154.14** | **6403** | **0.44** | **1** | **9348.14** | **0.51** |
|  | Homogeneity | 22183.63 | 6406 | 29.50 | 3 | 9371.63 | <0.01 |
| Physical bullying | Saturated | 14518.60 | 6388 | - | - | 1742.60 | - |
|  | Constrained saturated | 14538.27 | 6402 | 19.67 | 14 | 1734.27 | 0.14 |
|  | **Heterogeneity *** | **14542.81** | **6403** | **4.54** | **1** | **14542.81** | **0.03** |
|  | Homogeneity | 15015.98 | 6406 | 473.17 | 3 | 2203.982 | <0.01 |
| Verbal bullying | Saturated | 30786.18 | 6387 | - | - | 18012.18 | - |
|  | Constrained saturated | 30795.08 | 6401 | 8.91 | 14 | 17993.08 | 0.84 |
|  | **Heterogeneity *** | **30795.53** | **6402** | **0.44** | **2** | **17991.53** | **0.51** |
|  | Homogeneity | 30806.69 | 6405 | 11.17 | 3 | 17996.69 | 0.01 |
| Social manipulation | Saturated | 16970.19 | 6387 | - | - | 4196.19 | - |
|  | Constrained saturated | 16978.00 | 6401 | 7.80 | 14 | 4176.00 | .89 |
|  | **Heterogeneity *** | **16979.85** | **6402** | **1.85** | **1** | **4175.85** | **0.17** |
|  | Homogeneity | 17002.57 | 6405 | 22.72 | 3 | 4192.57 | <0.01 |
| Property damage | Saturated | 16024.29 | 6387 | - | - | - | - |
|  | Constrained saturated | 16029.83 | 6401 | 5.54 | 14 | 3227.83 | .98 |
|  | **Heterogeneity *** | **16029.95** | **6402** | **0.12** | **1** | **3225.95** | **0.73** |
|  | Homogeneity | 16096.07 | 6405 | 66.12 | 3 | 3286.07 | <0.01 |
| Sleep quality | Saturated | 12545.49 | 7444 | - | - | -2342.52 | - |
|  | Constrained Saturated | 12555.65 | 7458 | 10.16 | 14 | -2360.35 | 0.75 |
|  | Heterogeneity * | 12559.30 | 7459 | 3.65 | 1 | -2358.70 | 0.06 |
|  | **Homogeneity** | **12565.45** | **7462** | **6.16** | **3** | **-2358.55** | **0.10** |
| Insomnia | Saturated | 21998.32 | 7420 | - | - | 7158.32 | - |
|  | Constrained saturated | 22015.09 | 7434 | 16.77 | 14 | 7147.09 | 0.27 |
|  | **Heterogeneity *** | **22015.92** | **7435** | **0.83** | **1** | **7145.92** | **0.36** |
|  | Homogeneity | 22025.64 | 7438 | 9.73 | 3 | 7149.64 | 0.02 |

Note: Saturated model = estimates means and variances separately for twin 1 and twin 2, sex and zygosity groups; Constrained saturated = constrains means and variances across zygosity groups and for twins within the pair; Heterogeneity model = ACE paths estimated separately for males and females; Homogeneity model = ACE paths equated across males and females -2LL = negative 2 log likelihood; df = degrees of freedom; LRT = likelihood ratio *X^2^* test comparing the -2LL fit of each model to the -2LL fit of the saturated model; Δdf = difference in degrees of freedom comparing each model to the saturated model; AIC = Akaike’s Information Criterion (lower values reflect a better fit); p = *p*-value. *Best fitting model

Table S4: Fit statistics for bivariate ACE twin models

|  |  | Model Fit | | | | | |
| --- | --- | --- | --- | --- | --- | --- | --- |
| **Measures** | Model | -2LL | df | LRT | Δdf | AIC | P |
| Total bullying-Insomnia | Constrained Saturated | 44023.51 | 13829 | - | - | 16365.51 | - |
|  | Heterogeneity Model* | 44036.37 | 13832 | 12.86 | 3 | 16372.37 | 0.01 |
|  | Homogenetity Model | 44074.99 | 13841 | 38.63 | 9 | 16392.99 | <0.01 |
| Total bullying-Sleep quality | Constrained saturated | 34553.74 | 13853 | - | - | 6847.74 |  |
|  | Heterogeneity Model* | 34577.28 | 13856 | 23.54 | 3 | 6865.28 | <0.01 |
|  | Homogenetity Model | 34609.70 | 13865 | 32.42 | 9 | 6879.70 | <0.01 |

Note: Constrained Saturated model: estimates means and variances across sex and zygosity; Heterogeneity Model: different magnitude of genetic and environmental influences on variables and covariance between variables by sex. Homogeneity Model: genetic and environmental influences on and across variables equated across males and females. -2LL = negative 2 log likelihood; df = degrees of freedom; LRT = likelihood ratio *X^2^* test comparing the -2LL fit of each model to the -2LL fit of the saturated model; Δdf = difference in degrees of freedom comparing each model to the saturated model; AIC = Akaike’s Information Criterion (lower values reflect a better fit); p = *p*-value. *Best fitting model

Table S5: Harman’s single-Factor Test: Un-rotated principle component analyses

|  | Initial Eigenvalues | | | Extraction Sums of Squared Loadings | | |
| --- | --- | --- | --- | --- | --- | --- |
| Component | Total | Variance % | Cumulative % | Total | Variance % | Cumulative % |
| 1 | 8.57 | 21.42 | 21.42 | 8.57 | 21.42 | 21.42 |
| 2 | 5.43 | 13.57 | 35.00 | 5.43 | 13.57 | 35.00 |
| 3 | 1.89 | 4.73 | 39.72 | 1.89 | 4.73 | 39.72 |
| 4 | 1.63 | 4.07 | 43.79 | 1.63 | 4.07 | 43.79 |
| 5 | 1.41 | 3.52 | 47.31 | 1.41 | 3.52 | 47.31 |
| 6 | 1.29 | 3.23 | 50.54 | 1.29 | 3.23 | 50.54 |
| 7 | 1.22 | 3.05 | 53.59 | 1.22 | 3.05 | 53.59 |
| 8 | 1.15 | 2.88 | 56.47 | 1.15 | 2.88 | 56.47 |
| 9 | 1.10 | 2.75 | 59.22 | 1.10 | 2.75 | 59.22 |
| 10 | 0.99 | 2.46 | 61.68 |  |  |  |
| 11 | 0.95 | 2.38 | 64.06 |  |  |  |
| 12 | 0.88 | 2.20 | 66.26 |  |  |  |
| 13 | 0.85 | 2.12 | 68.38 |  |  |  |
| 14 | 0.84 | 2.09 | 70.47 |  |  |  |
| 15 | 0.79 | 1.99 | 72.45 |  |  |  |
| 16 | 0.77 | 1.91 | 74.36 |  |  |  |
| 17 | 0.73 | 1.81 | 76.18 |  |  |  |
| 18 | 0.67 | 1.66 | 77.84 |  |  |  |
| 19 | 0.62 | 1.56 | 79.40 |  |  |  |
| 20 | 0.61 | 1.52 | 80.92 |  |  |  |
| 21 | 0.57 | 1.43 | 82.35 |  |  |  |
| 22 | 0.57 | 1.41 | 83.77 |  |  |  |
| 23 | 0.55 | 1.39 | 85.15 |  |  |  |
| 24 | 0.54 | 1.34 | 86.49 |  |  |  |
| 25 | 0.48 | 1.19 | 87.69 |  |  |  |
| 26 | 0.46 | 1.15 | 88.83 |  |  |  |
| 27 | 0.45 | 1.12 | 89.95 |  |  |  |
| 28 | 0.44 | 1.11 | 91.05 |  |  |  |
| 29 | 0.39 | 0.98 | 92.03 |  |  |  |
| 30 | 0.38 | 0.95 | 92.98 |  |  |  |
| 31 | 0.37 | 0.93 | 93.91 |  |  |  |
| 32 | 0.35 | 0.88 | 94.79 |  |  |  |
| 33 | 0.33 | 0.82 | 95.61 |  |  |  |
| 34 | 0.31 | 0.78 | 96.39 |  |  |  |
| 35 | 0.30 | 0.75 | 97.14 |  |  |  |
| 36 | 0.28 | 0.69 | 97.83 |  |  |  |
| 37 | 0.27 | 0.67 | 98.50 |  |  |  |
| 38 | 0.23 | 0.58 | 99.08 |  |  |  |
| 39 | 0.22 | 0.54 | 99.61 |  |  |  |
| 40 | 0.15 | 0.39 | 100.00 |  |  |  |

Figure S1: Correlated factors solution of relationship between bullying-victimisation and sleep-quality


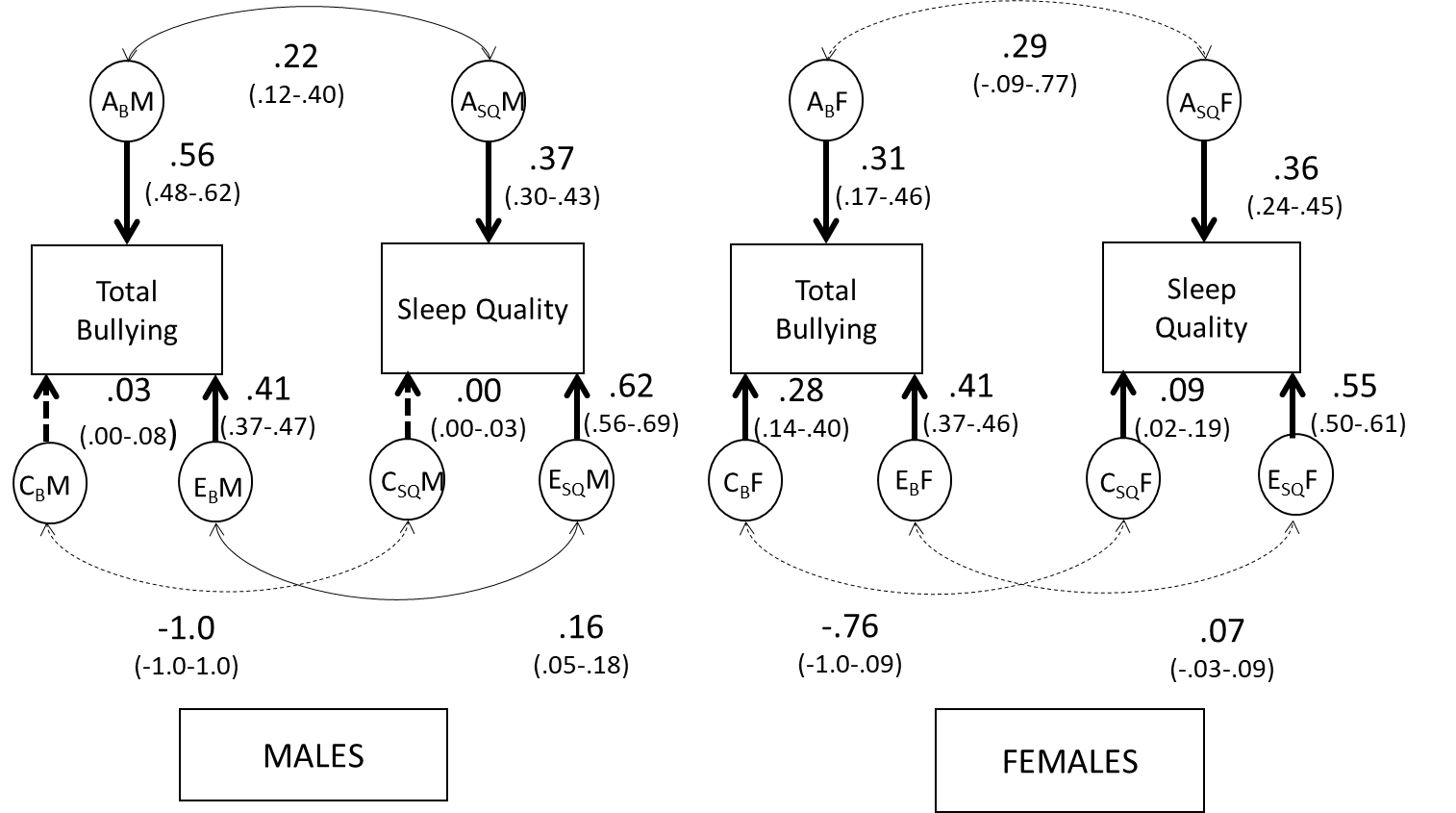


Figure S1a. A_B_M: Additive genetic influences bullying-victimisation males; A_SQ_M: Additive genetic influences sleep-quality males; C_B_M: Shared environmental influences bullying-victimisation males; C_SQ_M: Shared environmental influences sleep-quality males; E_B_M: Non-shared environmental influences bullying-victimisation males; E_SQ_M: Non-shared environmental influences sleep-quality males; A_B_F: Additive genetic influences bullying-victimisation females; A_SQ_F; Additive genetic influences sleep-quality females; C_B_F: Shared environmental influences bullying-victimisation females; C_SQ_F: Shared environmental influences sleep-quality females; E_B_F; Non-shared environmental influences bullying-victimisation females; E_SQ_F; Non-shared environmental influences sleep-quality females. Non-significant parameters indicated with doted lines.


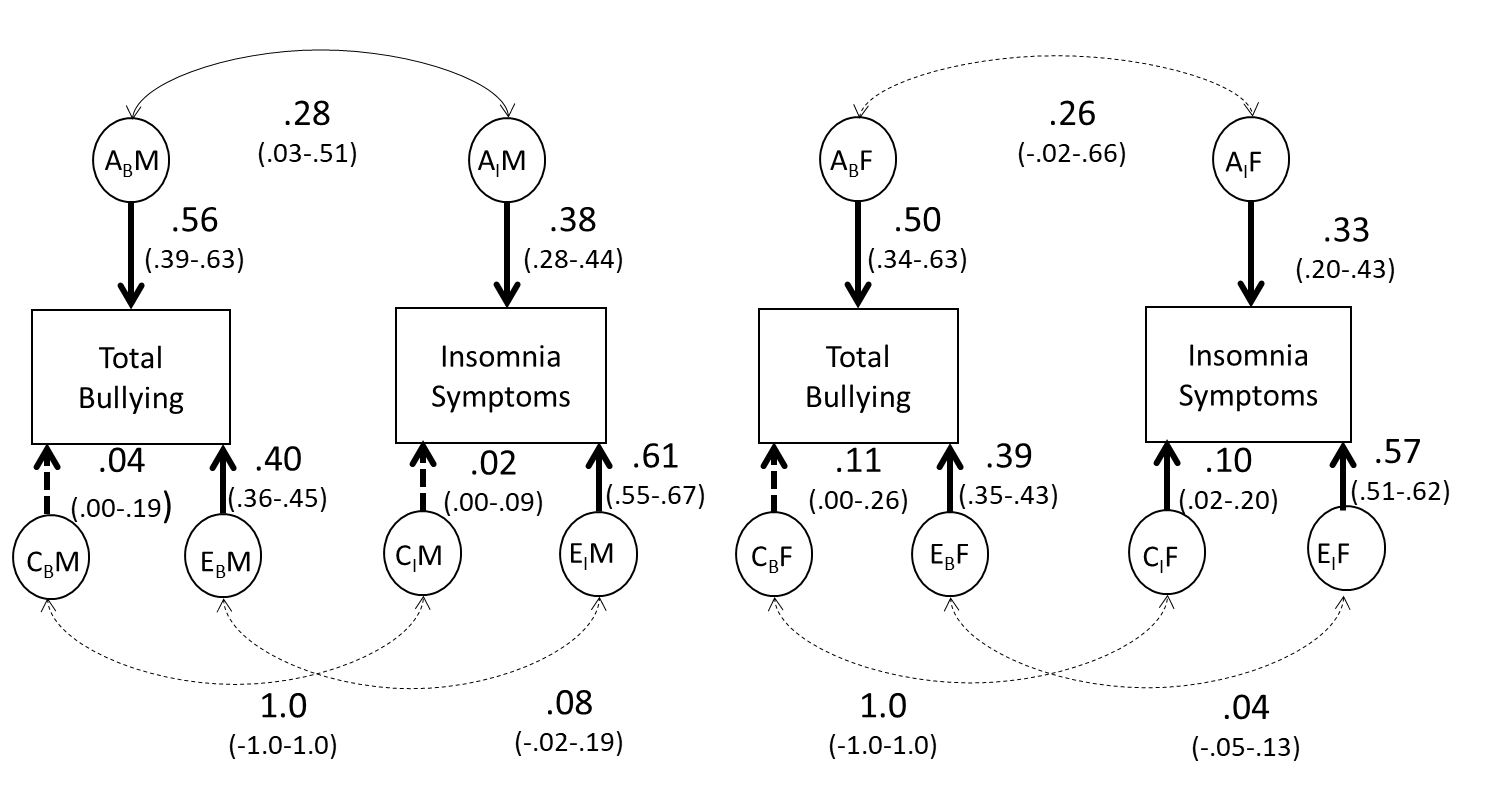


FEMALES

MALES

Figure S1b A_B_M: Additive genetic influences bullying-victimisation males; A_I_M: Additive genetic influences insomnia-symptoms males; C_B_M: Shared environmental influences bullying-victimisation males; C_I_M: Shared environmental influences insomnia-symptoms males; E_B_M: Non-shared environmental influences bullying-victimisation males; E_I_M: Non-shared environmental influences insomnia-symptoms males; A_B_F: Additive genetic influences bullying-victimisation females; A_I_F; Additive genetic influences insomnia-symptoms females; C_B_F: Shared environmental influences bullying-victimisation females; C_I_F: Shared environmental influences insomnia-symptoms females; E_B_F; Non-shared environmental influences bullying-victimisation females; E_I_F; Non-shared environmental influences insomnia-symptoms females. Non-significant parameters indicated with doted lines.

References only cited in supplementary material

Angold, A., Costello, E. J., Messer, S. C., Pickles, A., Winder, F., & Silver, D. (1995). Development of a short questionnaire for use in epidemiological studies of depression in children and adolescents. *International Journal of Methods in Psychiatric Research, 5*(4), 237-249.

Deater-Deckard, K., Dodge, K. A., Bates, J. E., & Pettit, G. S. (1998). Multiple risk factors in the development of externalizing behavior problems: group and individual differences. *Dev Psychopathol, 10*(3), 469-493.

Goodman, R. (1997). The Strengths and Difficulties Questionnaire: a research note. *J Child Psychol Psychiatry, 38*(5), 581-586.

Kaplan, E., Fein, D., Kramer, J., Delis, D., & Morris, R. (1999). WISC-III as a Process Instrument (WISC-III-PI). *New York: The Psychological Corporation.*

Matheny, A. P., Wachs, T. D., Ludwig, J. L., & Phillips, K. (1995). Bringing Order out of Chaos - Psychometric Characteristics of the Confusion, Hubbub, and Order Scale. *Journal of Applied Developmental Psychology, 16*(3), 429-444. doi: Doi 10.1016/0193-3973(95)90028-4

Neale, M. C., Roysamb, E., & Jacobson, K. (2006). Multivariate genetic analysis of sex limitation and G x E interaction. *Twin Research and Human Genetics, 9*(4), 481-489.
